# Assessment of foods for infants and toddlers in Australia against the World Health Organization’s Nutrient and Promotion Profile Model for food products for infants and young children

**DOI:** 10.1017/S136898002400171X

**Published:** 2024-10-04

**Authors:** Alexandra Chung, Sophia Torkel, Judith Myers, Helen Skouteris

**Affiliations:** 1 Department of Nutrition, Dietetics and Food, Monash University, Melbourne, Australia; 2 Monash Centre for Health Research and Implementation (MCHRI), Monash University, Melbourne, Australia; 3 Health and Social Care Unit, School of Public Health and Preventive Medicine, Monash University, Melbourne, Australia; 4 Nutrition and Dietetics, Faculty of Health, Charles Darwin University, Casuarina, NT, Australia; 5 Warwick Business School, University of Warwick, Coventry, UK

**Keywords:** Food marketing, Infant, Young children, Child nutrition, Food and nutrition policy

## Abstract

**Objective:**

Global public health agencies have recommended stronger regulation of food marketing to protect children’s diets. This study assessed commercial foods for infants and toddlers available in Australian supermarkets for compliance with the World Health Organization (WHO) Regional Office for Europe’s Nutrient and Promotion Profile Model: supporting appropriate promotion of food products for infants and young children 6–36 months in the WHO European Region (NPPM).

**Design:**

Dietitians assessed a sample of commercial foods for infants and toddlers against the composition, labelling and promotion requirements of the NPPM.

**Setting:**

Australia.

**Participants:**

Commercial foods for infants and toddlers (*n* 45) available in two major Australian supermarkets, purposely sampled across product categories and brands.

**Results:**

Fewer than one quarter (23 %) of the assessed products met all nutrient content requirements of the NPPM. No products met all of the labelling or promotional requirements. All products included at least one promotional marketing claim that was not permitted under the NPPM.

**Conclusions:**

The NPPM is useful to assess and monitor the nutritional composition and prevalence of marketing claims on commercial foods for infants and toddlers. Findings of noncompliance with the NPPM recommendations indicate an urgent need for stronger government regulation of the composition, labelling and marketing of commercial foods for infants and toddlers in Australia.

Unhealthy diets are a leading risk factor for the global burden of disease^(1)^. Early life is a critical time for the development of healthy dietary behaviours^(2)^ with adequate nutrition foundational for child development and the prevention of overweight and obesity^(3,4)^. Dietary patterns developed in infancy persist through the preschool years and into childhood^(5,6)^. Further, unhealthy diets in the first year of life contribute to socio-economic inequalities in excess weight among children^(7)^. There is therefore a window of opportunity in early childhood to promote healthy diets, reducing the risk of obesity and socio-economic inequalities in obesity.

Young children’s diets are increasingly reliant on commercial infant and toddler foods. This includes ready-made or processed foods for babies and toddlers up to 36 months of age that are sold in supermarkets, pharmacies and other stores. These foods commonly include purees and snack foods packaged in pouches, jars or boxes^(8)^. A review of infant and toddler diets across seven countries found between 40 and 60 % of infants aged 6–12 months consume commercial infant and toddler foods, with these foods making a significant contribution to total energy and sugar intake^(9)^. In Australia, one in two children aged up to 5 years consume commercial infant and toddler foods one or more days per week^(10)^. At the same time, commercial infant and toddler foods represent a growing segment of the grocery market, with increasing numbers of new products launched onto the market during the period 2003–2020^(11)^. In 2023, commercial baby food sales in Australia (including prepared baby food, cereals and snacks) generated USD$253·25 million in revenue^(12)^.

Commercial foods for infants and toddlers are often high in free sugars, including monosaccharides (such as glucose, fructose) and disaccharides (such as sucrose) added to foods and drinks by the manufacturer, as well as sugars naturally present in honey, syrups, fruit and vegetable juices, concentrates, purees, pastes and powders^(13,14)^. Fruit sugars are a particular problem in infant and toddler foods, which commonly contain sugars from fruit purees or fruit juice concentrate^(15,16)^. Yet these products are heavily promoted to children and their parents using a variety of marketing techniques, including visual appeals and health-related claims^(11,16,17)^

Food marketing impacts parents’ decision-making^(18)^ and children’s dietary behaviours, including food choices, preferences and purchase requests^(19)^. Marketing is pervasive on food and beverage product packaging with the use of colourful images and logos, popular children’s characters and health and nutrition claims^(20)^. On-pack marketing is a key component of cross-media marketing campaigns^(21)^ and a leading source of children’s exposure to unhealthy food marketing^(22)^. Parents are also targeted by front-of-pack marketing with health and nutrition claims commonly displayed on products for infants, toddlers and children^(17,23,24)^.

There are global efforts to reduce the harmful effects of food marketing on children’s diets^(25,26)^. Underpinned by a child rights approach, governments are being urged to implement regulations to protect children from the harmful effects of food marketing^(27)^. The WHO Regional Office for Europe has recently released the Nutrient and Promotion Profile Model: supporting the appropriate promotion of food products for infants and young children 6–36 months in the WHO European Region (NPPM)^(28)^. Developed in partnership with the University of Leeds, this model provides guidance on the composition and promotion of foods for infants and young children aged 6–36 months. In this model, the WHO recommends stronger regulation of the composition, labelling and promotion of foods for infants and toddlers to protect young children’s diets from the harms of commercial interests. The NPPM is accompanied by an online tool with a template that can be completed and uploaded to provide an assessment of commercially available products against the NPPM recommendations for product composition, labelling and promotion.

In Australia, compositional and labelling requirements of foods are regulated by The Australia New Zealand Food Standards Code. Foods for infants are addressed under Standard 2·9·2, which outlines compositional requirements such as limits on sugar and sodium, and minimum iron content in cereal-based foods, as well as labelling requirements, including age recommendations and vitamin and mineral content claims. Standard 1·2·7 outlines requirements for health claims that state or imply that a food has or may have a certain health effect, and nutrition content claims that refer to the presence or absence of macro or micronutrients (e.g. no added sugar, high in fibre, source of protein). However, Standard 1·2·7 does not apply to foods for infants. Furthermore, many of the claims commonly made on infant and toddler foods are not regulated under the Food Standards Code. This includes claims that make appeals to health and promote the product as ideal for young child feeding such as ‘natural’, ‘organic’, ‘no nasties’, ‘no preservatives’ and ‘for tiny hands’^(29)^. There is a distinct gap in the current regulation of commerical foods for infants and toddlers that allows manufacturers to influence children’s diets through the promotion of these products.

This study aimed to assess the nutritional composition, labelling, and promotion of commercial foods for infants and toddlers available in Australia’s major supermarkets against the requirements of the NPPM.

## Methods

### Data source

Data were collected from the websites of Australia’s two major supermarkets on December 14, 2021 using automated data extraction carried out by a private company for non-commercial purposes on behalf of members of the research team. Data included product images, product name, volume, pack size, price and nutritional composition for approximately 30 000 food and drink items. Using the supermarkets’ own classifications of baby and toddler food and after removing products with missing images or product information data, a dataset of 230 infant and toddler food products was created. For this study, nutrient content data were recorded directly from the data extraction output; details of package type, labelling and promotion were manually recorded from product images. Where possible, details from the data extraction output were used to populate the fields in the NPPM template. Missing data were identified in one of two ways: (1) online searches of supermarket websites (conducted between June 7 and July 2, 2023); (2) visits to physical supermarkets to locate products with package images captured on mobile phone for analysis. Supermarkets located in Chadstone and Clayton, Victoria, were visited by one author between June 28 and June 29, 2023.

### Study sample

In accordance with the protocol for a rapid evaluation outlined in the NPPM Product Evaluation Guide^(30)^, up to five products were selected from a range of brands across the fourteen NPPM subcategories. To do this, all products in our dataset were classified into NPPM product subcategories and then sorted by brand within each subcategory. Five products were then randomly selected from each subcategory ensuring at least one product was selected from each brand represented in the subcategory. Classification of food products to the relevant subcategories was performed by qualified dietitians with one author completing the initial allocation and a second author verifying the allocation of all products. In three subcategories, there were fewer than five products available in the data source, and in two categories (Ingredients and Drinks), no products were identified in the data source. The final analytical sample contained forty-five food items (Table 1) comprising products in assorted packaging, including pouches, jars, bowls, single-serve packets and sachets and boxes and packets containing multiple serves (Table 2).

**Table 1:** Number of products assessed in each of the WHO Nutrient and Promotion Profile Model product groups and subcategories

Product group	Code	Sub-category description	Number of products assessed
Dry cereals and starches	1	Dry or powdered cereal/starch to be eaten or cooked with milk or water	5
Dairy foods	2	Dairy-based foods, desserts, and cereals	5
Fruit and vegetable purees/smoothies and fruit desserts	3·1	Fruit-containing product, including breakfast/ dairy	5
	3·2	Vegetable only product	1
Savoury meals/ meal components: combinations of starches, vegetables, dairy and/or traditional protein Traditional protein sources include any meat, offal, poultry or fish	4·1	Food without protein or cheese named	5
	4·2	Food with cheese named but no protein	3
	4·3	Food with protein source not named first	5
	4·4	Food with protein source named first	5
	4·5	Protein source is only named food	0
Snacks and finger foods	5·1	Fruit	1
	5·2	Dry or semi-dry snacks and finger foods	5
Ingredients	6	Ingredients (e.g. olive oil, seasonings)	0
Confectionery	7	Confectionery	5
Drinks	8	Drinks (e.g. fruit juice, soft drinks)	0

**Table 2:** Package type according to food product group

Product group	Bowl	Jar	Multipack of single serves	Multi-serve box or sachet	Pouch with spout	Single-serve packet	Single-serve sachet
Dry cereals and starches (*n* 5)	0	0	0	5	0	0	0
Dairy foods (*n* 5)	0	1	0	0	4	0	0
Fruit and vegetable purees/smoothies and fruit desserts (*n* 6)	1	1	0	0	4	0	0
Savoury meals and meal components (*n* 18)	2	2	0	0	8	0	6
Snacks and finger foods (*n* 6)	0	0	1	0	0	5	0
Confectionery (*n* 5)	0	0	2	0	0	3	0

### Data analysis

The NPPM Product Evaluation Guide was applied to assess this sample of commercial foods for infants and toddlers^(30)^. The Guide includes a Microsoft Excel template to record nutrient data and on-pack promotions for infant and toddler food items for each product. The NPPM Guide offers an online calculator to automatically assess products against the NPPM guidelines; however, the calculator was offline for maintenance at the time of this study. Therefore, calculations were manually performed using Microsoft Excel to assess each product against the NPPM criteria and determine compliance with the NPPM guidelines.

## Results

The study sample consisted of forty-five items across six product groups: dry cereals and starches; dairy foods; fruit and vegetable purees; savoury meals; snacks and finger foods and confectionery. The assessment of each product against the requirements of the NPPM is discussed below and summarised in Table 3.

**Table 3:** Proportion of products meeting content, labelling and promotional requirements of the WHO Nutrient and Promotion Profile Model

	Requirements	Number of products subject to requirements	Number of products meeting requirement	% meeting requirement
Content and labelling requirements	Energy density (kcal/100 g)	40	21	53 %
	Na (mg/100 kcal)	40	38	95 %
	Total sugar (% E)	23	13	57 %
	Added free sugar or sweetener	40	34	85 %
	Total protein (g/100 kcal)	19	15	79 %
	Protein weight	10	6	60 %
	Total fat (g/100 kcal)	40	39	98 %
	No industrially produced trans fats	40	40	100 %
	Fruit content (% weight)	30	28	93 %
	Lower age label	40	37	93 %
	Upper age label	40	0	0 %
	Front-of-pack high in sugar label^*^	17	8	47 %
Promotional messages	No compositional, nutritional, health or marketing claims	45	0	0 %
	Product name clarity	45	18	40 %
	Ingredient list clarity	45	29	64 %
	Instructions not to consume soft foods via pack spout	16	3	19 %
	Suitable preparation instructions	5	5	100 %
	Promotion and protection of breastfeeding	45	0	0 %

* Seventeen products were required to be assessed against thresholds for the high in sugar label to be required; eight products were below the threshold.

### NPPM part A: content and labelling requirements

Of the forty assessed products subject to nutrient content requirements, less than one-quarter (23 %) met all nutrient content requirements. Of the forty products subject to energy density requirements, over half (53 %) met these requirements. The *Snacks and finger foods* product group was subject to an upper limit for energy density (≤50 kcal per serve), and all products in this product group met this requirement. Four product groups were subject to a minimum requirement for energy density, and each of these product groups contained products which had insufficient energy density, with products being between 0·1 % and 48 % below the minimum energy density requirement. The one product in the subcategory *3.2 Vegetable only products* was subject to the requirement to have no more than 25 % added water and contained 42·8 % added water, exceeding the requirement by 71 %.

Of the forty products subject to sodium content requirements (≤50 mg/100 kcal, or ≤100 mg/100 kcal if cheese is named), all but two of the products (95 %) met the requirements for sodium content. The two products which exceeded the sodium content threshold were in subcategories *Food without protein or cheese named* and *Dry or semi-dry snacks and finger foods*. These products were 38 % and 7 % above the upper limit for sodium content, respectively.

Of the twenty-three products subject to total sugar limits (≤15 % energy from sugar), over half (57 %) met these requirements. However, among products packaged in pouches with a spout, only 25 % were within the limits for total sugar. The ten products which exceeded the sugar content threshold were between 16 % and 97 % above the maximum sugar content requirement. Of the seventeen products subject to evaluation for the need to carry a ‘high in sugar’ flag, over half (53 %) would be required to carry a ‘high in sugar’ flag. Of the forty products subject to the requirement to have no added free sugars or sweeteners, the majority (85 %) met this requirement. The products containing added free sugar were in the subcategories *Dairy-based foods, desserts, and cereals* and *Fruit-containing product, including breakfast/ dairy.* Of the thirty products subject to fruit content requirements, the majority (93 %) met these requirements. One product in the subcategory *Food with cheese named but no protein* contained added fruit but did not report on the amount of fruit added, while one product in the *Fruit* subcategory contained 99·5 % fruit compared to the requirement to contain 100 % fruit.

Of the nineteen products subject to total protein content requirements, over three-quarters (79 %) met these requirements. The four products which did not meet protein content requirements were in the subcategory *Food without protein or cheese named* and were between 17 % and 39 % below the minimum protein content requirement. Of the ten products subject to minimum protein weight requirements, over half (60 %) met these requirements. The four products which did not meet protein weight requirements were between 13 % and 65 % below the minimum protein weight requirement.

Of the forty products subject to total fat content limits, all but one of the products (98 %) met the requirements for total fat content. The one product which exceeded the threshold was in the category *Dry or semi-dry snacks and finger foods* and contained 16 % more fat than the maximum permitted fat content. No products contained industrially produced trans fats.

### NPPM part B: promotional messages

All forty-five products displayed at least one nutrition, marketing or health claim, therefore none of the products in this sample met the NPPM promotional message requirements of ‘no compositional, nutritional, health or marketing claims’. Over half of the products (60 %) did not fulfil the requirements for product name clarity. Reasons for not fulfilling these requirements included listing the ingredients in an order other than descending order. One-third of products (36 %) did not meet the NPPM requirements for ingredient list clarity. Reasons for not meeting the NPPM requirements for ingredient list clarity included missing information on the amount of added water or fruit.

Sixteen products were packaged in pouches, including savoury meals (*n* 8), fruit and vegetable purees (*n* 4), and dairy foods (*n* 4). Under NPPM requirements, these are subject to provide a suggestion to serve the product from a spoon or bowl. Less than one-fifth (19 %) of pouch products met the requirement to provide instructions not to consume the product directly from the spout, and none included an upper-age label. In addition, some products with spouts included non-permitted claims promoting the convenience of the product as an ‘on-the-go’ snack. Of all five products which required preparation, all provided preparation requirements which were classified as suitable according to the NPPM.

Analysis of compliance with NPPM requirements according to package type found that overall compliance with NPPM requirements was similar across all package types. For example, 55 % of NPPM criteria were met for pouch products, 56 % of criteria were met for products in a bowl and 60 % of criteria were met by products in jars. Detailed results are provided in, Supplementary File 1.

No products met all NPPM requirements in relation to protecting and promoting breastfeeding. The NPPM specifies that ‘all products must include a statement on the importance of continued breastfeeding for up to 2 years or beyond and the importance of not introducing complementary feeding before 6 months of age’, but no products in this study included such a statement. In addition, the labels of three of the products were incongruent with breastfeeding guidelines by including a statement that the product was suitable for infants from 4 months of age. No products provided information on upper age limits on the package.

## Discussion

This study assessed a sample of forty-five commercial foods for infant and toddlers available in Australian supermarkets against the NPPM guidelines for commercial foods for infants and young children. All food products assessed in this study failed to meet the NPPM promotional requirements by including marketing features not permitted under the NPPM guidelines, and fewer than one-quarter of assessed products met all of the nutritional composition requirements of the NPPM.

Added sugar limits were exceeded in almost half the products assessed. The addition of free sugars is of particular concern due to the risks of excess weight gain, poor dental health and development of a preference for sweet foods^(13,31)^. The use of fruit as an added sugar is common in infant and toddler foods, which frequently contain sugars from fruit purees or fruit juice concentrate^(15,16)^. The presence of sugar in infant and toddler food is a concern globally, with a study examining product data in twenty-seven European countries finding that 24·4 % of products contain free sugars and 38·5 % contain at least one sugar-contributing ingredient (e.g. fruit purees)^(32)^.

The definition of ‘added sugar’ is an ongoing source of discussion, particularly in Australia and New Zealand, where there is no agreed definition^(33)^. Further, in Australia, there is currently no requirement for manufacturers to declare the amount of added sugar in a food. There is broad support for clear added sugar labelling on food packages^(34)^, yet under the current system, sugar labelling on infant and toddler foods is misleading^(29,35)^.

An important feature of foods for infants and toddlers is to provide adequate nutrition for growth^(36)^. However, only around half of the products assessed in this study met minimum energy density requirements. Australia’s Infant Feeding Guidelines recommend the introduction of nutritious solid foods around the age of 6 months alongside continued breastfeeding^(36)^. This is echoed in UNICEF guidelines that recommend nutritious, age-appropriate foods during the complementary feeding period and avoid foods with low nutritional value or with added sugars^(37)^. The increasing reliance on commercial foods for infants and toddlers places young children at risk of not meeting nutritional requirements for growth and development.

Products in pouches with spouts included suggestions to serve the product from a spoon or bowl but failed to provide explicit instructions not to consume via the spout. Despite the convenience of a spout, consuming purees from a spout can interfere with oral-motor development, which relies on chewing and swallowing food with increasingly complex textures^(38)^. Pouches also reduce the opportunity to experience the look, smell and feel of foods and have been associated with fussy eating^(39)^. Evidence also shows that products in pouches are nutritionally poor, often high in free sugars, lack iron fortification and are heavily promoted by marketing that is misleading^(35)^. Regular monitoring of infant and toddler diets is important to provide insights into the extent to which young children’s diets include commercially available foods including those packaged in pouches, and further our understanding of the impacts of these products on children’s diets.

No products met the NPPM requirements to promote and protect breastfeeding. The WHO recommends exclusive breastfeeding for the first 6 months of life, followed by continued breastfeeding for up to 2 years or beyond, with appropriate complementary foods^(40)^. Similarly, Australia’s Infant Feeding Guidelines recommend that infants are exclusively breastfed until around 6 months of age, with solid foods introduced at around 6 months but not before 4 months, with continued breastfeeding until 12 months of age and beyond, for as long as the mother and child desire^(36)^. Products failed to meet the NPPM requirements to state the importance of breastfeeding and some products were promoted as ‘suitable for 4+ months’ in direct conflict with the intentions of optimal infant feeding advice. By failing to promote the benefits of breastfeeding and providing feeding advice that directly contradicts best practice, manufacturers are undermining the importance of breastfeeding.

All forty-five products included at least one health or nutrition claim, and all therefore failed to meet the NPPM requirement of ‘no compositional, nutritional, health or marketing claims’. Research has previously shown that nutrition and product composition claims are prolific on infant and toddler food packages. For example, assessment of on-pack claims on commercial baby foods (for infants up to 12 months) in UK supermarkets found extensive use of composition, nutrient and marketing claims across almost all products, while health claims such as those implying benefits for health or growth were observed to a much lesser extent^(23)^. An Australian study found claims appealing to health or nutrition present on every product in a sample of 230 infant and toddler foods and additionally found up to 15 unique marketing features present on the front-of-pack^(17)^. An audit of health and nutrition claims on infant and toddler food packaging in Australia found pervasive use of health-related appeals including statements and images that imply a product is ‘natural’, and developmental claims to promote products as ‘better-for-you’^(16)^. In Taiwan, a study of infant and toddler foods found composition, health and nutrient claims frequently appeared on the front-of-pack, yet these products were no more healthy than similar products without claims^(24)^. These findings suggest that infant and toddler food packages may be misleading parents by making extensive health-related appeals including claims about product composition and nutritional value.

The NPPM provides guidance on written health and nutrition claims but does not consider visual marketing cues or other marketing features that may appeal to children. Child-directed marketing is a powerful technique used by the food industry to influence children’s preferences, choices, purchase requests and consumption^(19)^. Based on the evidence of extensive child-appeal marketing on infant and toddler food products^(17,41)^, the NPPM will be best considered *alongside* other international guidance (such as the WHO Set of Recommendations on the marketing of foods and non-alcoholic beverages to children^(25)^ and Policies to protect children from the harmful impact of food marketing^(26)^) to ensure children’s diets are adequately protected against the harms of marketing in all its forms.

Amidst global concerns that food marketing is driving consumption of commercial infant and toddler foods and displacing intake of fresh foods^(42)^, there is a need for detailed policy guidance to ensure foods marketed and sold for infants and toddlers support optimal nutrition and child development. Policy guidance is necessary to ensure governments act to protect population diets from commercial interests which play a powerful role in shaping food systems and food environments^(43)^. As legislation is developed and implemented, policy guidance will also be required for ongoing monitoring and compliance to ensure policies have the intended effects in protecting children’s diets and to hold food companies to account^(44)^.

### Limitations

These findings should be considered within the limitations of the research methods. This rapid evaluation had a small sample size of forty-five products. This provides insight into the nutritional composition and marketing of infant and toddler foods in Australia but does not represent all products in the food supply. Because there were small numbers in each product category, it is unknown whether the sample had adequate statistical power to detect differences in marketing features between groups. However, the study methods are in accordance with the protocol recommended in the NPPM product evaluation guide and as such allow for replication across larger samples and to monitor trends over time.

### Implications

The NPPM is an important tool to guide policy reform to support optimal nutrition and development for infants and young children. The tool aims to reduce inappropriate promotion of foods for infants and young children including any promotion that interferes with breastfeeding, contributes to obesity and non-communicable disease, creates a dependency on commercial products, or is otherwise misleading. The NPPM model and accompanying analysis tool has wide application for monitoring the composition, labelling and promotion of commercial foods for infants and toddlers over time and compliance with international and local standards. Using a sample of commercially available infant and toddler food products in the Australian food supply, this study demonstrates the utility of the NPPM tool and provides evidence of the urgent need to improve the composition, labelling and promotion of commercial foods for infants and young children in Australia. The tool can be easily accessed and used in food systems monitoring, public health nutrition research and advocacy for regulation to improve the composition, labelling and promotion of foods for infants and young children.

### Conclusion

Commercial foods for infants and toddlers are heavily marketed to parents as an ideal choice. Yet this study found that many products on the Australian market failed to meet the requirements of the NPPM. All infant and toddler food products assessed in this study included marketing claims not permitted under the NPPM guidelines, and only one in four products met all of the nutritional composition requirements of the NPPM. Whilst most products met nutritional requirements such as sodium limits, almost half exceeded total sugar limits. This demonstrates an urgent need for governments to set higher standards for the composition, labelling and promotion of foods for infants and young children. The NPPM is an important addition to the suite of resources available to public health advocates, practitioners and governments to support the design and implementation of policies that protect children’s diets and promote optimal infant and young child feeding.

## Supporting information

Chung et al. supplementary material Chung et al. supplementary material

## Supplementary material

For supplementary material accompanying this paper, visit https://doi.org/10.1017/S136898002400171X
