# Supplementary material for: Assessment of foods for infants and toddlers in Australia against the World Health Organization’s Nutrient and Promotion Profile Model for food products for infants and young children
Source: Public Health Nutr. 2024 Oct 4;27(1):e201. doi: 10.1017/S136898002400171X (PMC11504510; doi:10.1017/S136898002400171X)
Supplement: Chung et al. supplementary material [file S136898002400171Xsup001.docx]

Table S1. Products meeting World Health Organization’s Nutrient and Promotion Profile Model requirements according to package type

|  | Requirements | Bowl (n = 3) | Jar (n = 4) | Multipack of single serves (n = 3) | Multi-serve box or sachet (n = 5) | Pouch with spout (n = 16) | Single serve packet (n = 8) | Single serve sachet (n = 6) |
| --- | --- | --- | --- | --- | --- | --- | --- | --- |
| Content and labelling requirements | Energy density (kcal/100 g) | 1/3 (33%) | 3/4 (75%) | 1/1 (100%) | 0/5 (0%) | 8/16 (50%) | 5/5 (100%) | 3/6 (50%) |
|  | Sodium (mg/ 100 kcal) | 2/3 (67%) | 4/4 (100%) | 1/1 (100%) | 5/5 (100%) | 16/16 (100%) | 4/5 (80%) | 6/6 (100%) |
|  | Total sugar (% E) | 0/2 (0%) | 1/2 (50%) | N/A | N/A | 2/8 (25%) | 5/5 (100%) | 5/6 (83%) |
|  | Added free sugar or sweetener | 3/3 (100%) | 3/4 (75%) | 1/1 (100%) | 5/5 (100%) | 11/16 (69%) | 5/5 (100%) | 6/6 (100%) |
|  | Total protein (g/100 kcal) | 0/2 (0%) | 2/2 (100%) | N/A | 1/1 (100%) | 7/8 (88%) | N/A | 5/6 (83%) |
|  | Protein weight | N/A | 2/2 (100%) | N/A | N/A | 3/5 (60%) | N/A | 1/3 (33%) |
|  | Total fat (g/100 kcal) | 3/3 (100%) | 4/4 (100%) | 1/1 (100%) | 5/5 (100%) | 16/16 (100%) | 4/5 (80%) | 6/6 (100%) |
|  | No industrially produced trans fats | 3/3 (100%) | 4/4 (100%) | 1/1 (100%) | 3/5 (100%) | 16/16 (100%) | 5/5 (100%) | 6/6 (100%) |
|  | Fruit content (% weight) | 3/3 (100%) | 3/3 (100%) | 0/1 (0%) | 5/5 (100%) | 11/12 (92%) | N/A | 6/6 (100%) |
|  | Lower age label | 3/3 (100%) | 3/4 (75%) | 1/1 (100%) | 3/5 (60%) | 16/16 (100%) | 5/5 (100%) | 6/6 (100%) |
|  | Upper age label | 0/3 (0%) | 0/4 (0%) | 0/1 (0%) | 0/5 (0%) | 0/16 (0%) | 0/5 (0%) | 0/6 (0%) |
|  | Front-of-pack high in sugar label | 1/1 (100%) | 1/2 (50%) | 0/1 (0%) | 5/5 (100%) | 1/8 (13%) | N/A | N/A |
| Promotional messages | No compositional, nutritional, health or marketing claims | 0/3 (0%) | 0/4 (0%) | 0/3 (0%) | 0/5 (0%) | 0/16 (0%) | 0/8 (0%) | 0/6 (0%) |
|  | Product name clarity | 2/3 (67%) | 1/4 (25%) | 1/3 (33%) | 3/5 (60%) | 5/16 (31%) | 3/8 (38%) | 3/6 (50%) |
|  | Ingredient list clarity | 2/3 (67%) | 2/4 (50%) | 3/3 (100%) | 2/5 (40%) | 6/16 (38%) | 8/8 (100%) | 6/6 (100%) |
|  | Instructions not to consume soft foods via pack spout | N/A | N/A | N/A | N/A | 3/16 (19%) | N/A | N/A |
|  | Suitable preparation instructions | N/A | N/A | N/A | 5/5 (100%) | N/A | N/A | N/A |
|  | Promotion and protection of breastfeeding | 0/3 (0%) | 0/4 (0%) | 0/3 (0%) | 0/5 (0%) | 0/16 (0%) | 0/8 (0%) | 0/6 (0%) |
|  | % of criteria met ^a^ | 56% | 60% | 48% | 59% | 55% | 61% | 68% |

^a^ Calculated as follows: numerator is the sum of numerators in above rows; denominator is the sum of denominators in above rows.
